# Microencapsulated Caraway Essential Oil Affects Initial Growth of Maize Cultivars

**DOI:** 10.3390/molecules26165059

**Published:** 2021-08-20

**Authors:** Katarzyna Możdżeń, Agnieszka Krajewska, Jan Bocianowski, Beata Jop, Agnieszka Synowiec

**Affiliations:** 1 Institute of Biology, Pedagogical University of Krakow, Podchorążych 2, 30-084 Kraków, Poland; katarzyna.mozdzen@up.krakow.pl; 2 Institute of Natural Products and Cosmetics, Lodz University of Technology, 90-924 Łódź, Poland; agnieszka.krajewska@p.lodz.pl; 3 Department of Mathematical and Statistical Methods, Poznań University of Life Sciences, Wojska Polskiego 28, 60-637 Poznań, Poland; jan.bocianowski@up.poznan.pl; 4 Department of Agroecology and Crop Production, Faculty of Agriculture and Economics, The University of Agriculture in Krakow, Mickiewicza 21, 31-120 Kraków, Poland; beata.jop@urk.edu.pl

**Keywords:** biocidal effect, chlorophyll fluorescence, dose–response test, phytotoxicity, relative chlorophyll content

## Abstract

Caraway (*Carum carvi* L.) essential oil is a candidate for botanical herbicides. A hypothesis was formulated that the sand-applied maltodextrin-coated caraway oil (MCEO) does not affect the growth of maize (*Zea mays* L.). In the pot experiment, pre-emergence application of five doses of MCEO was tested on four maize cultivars up to the three-leaf growth stage. The morphological analyses were supported by the measurements of relative chlorophyll content (SPAD), two parameters of chlorophyll *a* fluorescence, e.g., Fv/Fm and Fv/F0, and fluorescence emission spectra. The analyzed MCEO contained 6.5% caraway EO with carvone and limonene as the main compounds, constituting 95% of the oil. The MCEO caused 7-day delays in maize emergence from the dose of 0.9 g per pot (equal to 96 g m^−2^). Maize development at the three-leaf growth stage, i.e., length of roots, length of leaves, and biomass of shoots and leaves, was significantly impaired already at the lowest dose of MCEO: 0.4 g per pot, equal to 44 g m^−2^. A significant drop of both chlorophyll *a* fluorescence parameters was noted, on average, from the dose of 0.7 g per pot, equal to 69 g m^−2^. Among the tested cultivars, cv. Rywal and Pomerania were less susceptible to the MCEO compared to the cv. Kurant and Podole. In summary, maize is susceptible to the pre-emergence, sand-applied MCEO from the dose of 44 g m^−2^.

## 1. Introduction

Essential oils (EOs) can effectively inhibit germination and early growth of weeds, and for that reason, they could be utilized in the future as so-called botanical herbicides [1,2]. According to research results, those EOs that are rich in oxygenated monoterpenes display significant allelopathic effects [3]. One of them is caraway (*Carum carvi* L.) EO [3,4]. Caraway is an annual or biennial herb in the Apiaceae botanical family [5], native to western Asia, Europe, and North Africa. Caraway is commonly cultivated in Europe, i.e., The Netherlands, Germany, Finland, Czech Republic, Slovakia, and Hungary [6,7,8]. The species is characterized by a high phenotypic variability [6,9].

The caraway EO is steam-distilled from the achenes of caraway. The oil is a clear, colorless, or yellow liquid with a pleasant aroma and spicy flavor. The content of oil in the achenes is highly variable [10], e.g., 3.2–5.2% in Polish cultivars [11] and 3.31–4.06 in the Serbian ones [12]. Two compounds dominate the EO, i.e., carvone, an oxygenated monoterpene, and limonene, a monoterpene hydrocarbon, constituting together 93.3–98.1% of all oil compositions. In contrast, the remaining compounds, i.e., carvacrol, α-pinene, γ-terpinene, linalool, carvenone, and p-cymene, are present only in traces [5,13,14,15]. In Polish cultivars, carvone and limonene are found in quantities of 55.4–71.6% and 25.0–40.3% of the oil, respectively [11].

Caraway EO and carvone display several biological activities, e.g., antibacterial or fungicidal [14,16,17] and herbicidal, inhibiting germination and early growth of weeds such as *Avena fatua* L., *Bromus secalinus* L., *Amaranthus retroflexus* L., *Centaurea cyanus* L., and *Matricaria chamomilla* L. [3,18]. The herbicidal property of caraway EO could be used in the future by applying this oil to the soil as a pre-emergence weed control agent. However, to apply it precisely, the EO should be well formulated with a carrier to improve its application [19]. One method to do so is to encapsulate the EO, which means to coat it with different solid carriers [20]. Encapsulation also extends the biological activity of the EO by its slow release [21]. A common carrier of microencapsulated EOs is maltodextrin, a polysaccharide with high solubility in water and low viscosity [22,23], usually applied in the process of dry-spraying [23]. The allelopathic effect of the microencapsulated caraway oil with maltodextrin as a carrier (MCEO) was verified in the previous experiments. In pot experiments, the MCEO mixed with peat and sand, at a dose of 200 g m^−2^, as a pre-emergence agent, significantly inhibited the initial growth of two weeds–lambs quarters (*Chenopodium album* L.) and barnyard grass (*Echinochloa crus-galli* (L.) P. Beauv.), but also maize (*Zea mays* L. ‘Wilga’) [24]. The inhibiting effects of two doses of 7.5% MCEO of 50 and 100 g m^−2^ were further confirmed in a two-year-long field experiment with maize carried out on clayey brown soil. We showed that the higher dose of MCEO reduced the number of maize plants and cobs per 1 m^2^ by 17% and 21%, respectively, compared to the non-treated control [25].

Early detection of phytotoxic action of the EOs is important in assessing their suitability as botanical herbicides and in testing their safety on crops. Photosynthesis is one of the most sensitive physiological processes affected by different types of stresses [26]. The early stress response could be detected by measuring the disturbances in the light phase of photosynthesis [27]. This process mainly occurs in chloroplasts that absorb light energy (especially blue and red light) by molecules of the assimilation pigments mainly in the photosystem II, which trigger further photochemical reactions [28,29]. The excess of energy is emitted as a flush of chlorophyll *a* fluorescence. In a few previous works, it was proved that the phytotoxic action of various EOs could be assessed early by this method, by measuring the Fv/Fm parameter, a sensitive plant-stress indicator, referring to the maximum primary yield of the photochemistry of photosystem II [27], i.e., for clove oil and its main compounds [30], leaf-applied caraway and peppermint oils [4] or mint and cinnamon EOs [31]. The chlorophyll fluorescence method can be supported by measuring chlorophyll fluorescence emission at different wavelengths [32,33], which can also be successfully applied to stress detection in plants [34,35].

Studying the herbicidal effects of essential oil on crops is crucial for selecting the crop-safe oil, its dose [36] and a safe method for the EO application. Previous experiments showed that the phytotoxic effects of soil-applied and maltodextrin-coated essential oils of caraway or peppermint depend on the growth medium [24,37], dose of oil, and maize cultivar [37]. For those reasons, in this dose–response experiment, we tested how different doses of caraway EO, encapsulated in maltodextrin and pre-emergence sand-applied, will affect the initial growth of four different maize (*Zea mays* L.) cultivars.

## 2. Results

### 2.1. Chemical Composition of the Microencapsulated Caraway Oil

The analyzed microcapsules contained 6.5% of the caraway oil. There were 99% compounds isolated by GC-MS in the oil, among which carvone and limonene dominated, constituting together 95% of the oil (Table 1). The average content of limonene in the oil was 15.2% and carvone–79.9%. The oil also contained 1.4% of dihydrocarvone but lacked *trans*-carveol.

### 2.2. Emergence, Growth, and Chlorophyll a Fluorescence Parameters of Maize Cultivars

In the control treatments, maize started to emerge 4–5 days after sowing. Maize cvs Pomerania (PM), Kurant (KU), and Podole (PO) emerged in the same time regime as controls up to the dose of 0.9 g per pot (equal to 96 g m^−2^), whereas cv. Rywal (RY) up to the dose of 0.7 g pot (equal to 69 g m^−2^). The emergence of maize cv. RY was delayed by seven days, compared to the control at doses 0.9–1.2 g pot (equal to 96–127 g m^−2^). At the dose of 1.8 g pot (192 g m^−2^), the cvs. PM and RY did not emerge.

The results of the MANOVA indicated that all the cultivars (Wilk’s λ = 0.00004; F = 179.60; *p* < 0.0001), MCEO doses (Wilk’s λ = 0.00000019; F = 155.81; *p* < 0.0001) and cultivar × dose interactions (Wilk’s λ = 0.000000001; F = 35.88; *p* < 0.0001) were significantly different concerning all of the thirteen quantitative traits. ANOVA indicated that the main effects of dose per pot were significant for all studied traits (Table 2). The main effects of cultivars were significant for all the traits except for shoot and fresh root mass (Table 2). The effects of cultivar × dose per pot interaction were statistically significant for all traits except fresh shoot mass (Table 2).

Different doses of MCEO significantly affected the growth of maize cultivars at the three-leaf stage. Only at the lowest dose of MCEO, i.e., 0.4 g per pot (equal to 44 g m^−2^), the tested biometric parameters of all maize cultivars were similar to that of the controls (Table 3). At higher doses of MCEO, discrepancies in development between particular maize cultivars were noted. Specifically, almost all biometrical traits of cv. RY up to a dose of 0.7 g MCEO per pot (equal to 69 g m^−2^) was similar to the control, except for the root length, by 20% lower. In the case of the other three cultivars, at a dose of 0.7 g per pot, a significant decrease of plants length and biomass accumulation by 20–70%, compared to the control, was noted. Cultivar ‘Kurant’ (KU) was the only cultivar that grew at the highest applied dose of MCEO (1.8 g per pot, equal to 192 g m^−2^), although its growth was by 93–95% inhibited, compared to the control. 

The length of maize roots was the most affected biometrical trait of maize; a significant decrease of roots length, by 10%, compared to the control, was noted already at the lowest dose of MPEO. In the other traits, a significant drop of their values was noted at a dose of 0.7 g MCEO per pot (equal to 69 g m^−2^).

The values of SPAD and the chlorophyll *a* fluorescence parameters differed among maize cultivars treated with the MCEO (Table 4). Although the average values of SPAD and Fv/Fm for all the cultivars showed a significant drop already from the lowest dose of MCEO (0.4 g per pot, equal to 44 g per m^−2^), there were significant differences between particular cultivars. The SPAD and the fluorescence parameters (Fv/Fm, Fv/F0, F690/F735, F450/F690, and F450/F735) dropped significantly from the lowest dose of MCEO for cv. PM only. In the case of cv. PO a significant drop of SPAD was noted from the lowest dose of MCEO and was correlated with increased F450/F735 parameter values. At the same time, the Fv/Fm parameter for cv. PO dropped significantly only from the dose of 0.9 g per pot (equal to 96 g m^−2^). For cv. KU, a significant drop of SPAD, Fv/Fm, and Fv/F0 was noted only from 0.9 g per pot (96 g m^−2^). In the case of cv. RY, the SPAD value dropped only at a dose of 0.7 g per pot (equal to 69 g m^−2^), and the Fv/Fm parameter was significantly lower than the control for doses 0.7–1.8 g per pot (69–192 g m^−2^).

The Pearson’s correlation analysis revealed that the correlation coefficients were statistically significant (at 0.05 level) between all pairs of traits (Table 5). Two parameters of chlorophyll fluorescence, i.e., Fv/Fm and Fv/F0, were highly correlated with each other (0.994) and correlated with the biometrical traits and relative chlorophyll content in the leaves (SPAD). The fresh root mass was also strongly correlated with the leaves and root length (0.95–0.96). The four emission spectra, i.e., F690/F735, F450/F690, F450/F735, and PSIIA/C, were highly correlated with each other but less with the other traits. The lowest correlation coefficient was observed between root length and the F690/F735 spectrum (0.41).

**Figure 1:**
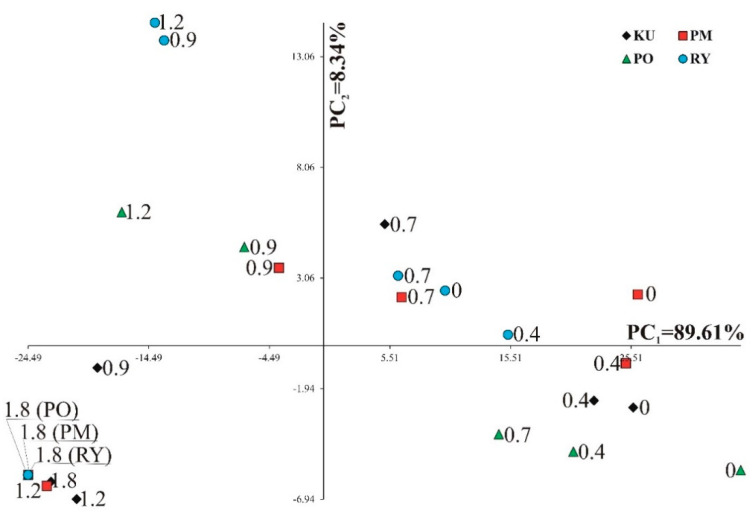
The distribution of 24 combinations of cultivars times the dose per pot in the two first principal components was calculated based on 13 analyzed traits of maize.

The PCA analysis helped to show a distribution of cultivars and MCEO doses to the two main principal components. The values for the first two principal components were also significant and accounted jointly for 97.95% of the whole variation (Figure 1). Significant positive linear relationships with the first principal component were found for all thirteen observed traits. The second principal component had a significant positive correlation with SPAD, F690/F735, and PSIIA/C. The PCA analysis showed the clustering of doses but also the cultivar × doses. At lower doses of MCEO, both main factors played a role; cv. Rywal (RY) and Pomerania (PM) at doses 0–0.7 g per pot performed the best. The cultivars clustered at the two highest doses of MCEO (1.2–1.8 g per pot, equal to 127 and 192 g m^−2^).

Based on the PCA and Pearson’s correlation coefficients the following hierarchy of individual traits on the MCEO effect on maize was drawn: length of 3rd leaf > length of 1st leaf > length of root > length of 2nd leaf > shoot fresh mass > root fresh mass > Fv/F0 > Fv/Fm > SPAD > F450/F690 > F450/F735 > PSIIA/C > F690/F735.

## 3. Discussion

The composition of analyzed caraway essential oil was dominated by carvone and limonene, which is typical for this oil [7]. The content of these two components corresponds to the literature data [38] but differs with the European Pharmacopoeia [39], as the oil contained ca. 50% less of limonene and 23% higher content of carvone. Other typical ingredients were also found in the tested oil, such as dihydrocarvone, but at the same time, the oil did not contain carveol and dihydrocarveol as well as α-pinene. The microencapsulation process (spray drying) might cause these differences, as was also found in dry spraying of peppermint oil [40]. As a (+)-carvone enantiomer, a major compound of caraway essential oil, carvone could be mainly responsible for the biocidal effects of the oil. It is a highly phytotoxic compound [41], i.e., it completely inhibits germination of *Lolium rigidum* (Gaud.) at 160 nL cm^−3^ [42] and *Setaria verticillata* ((L.) P. Beauv) at 80 nL cm^−3^ [43]. Moreover, (+)-carvone is also known for its insecticidal abilities against *Diabrotica virgifera* (LeConte) [44] and acaricidal against *Rhipicephalus microplus* (Canestrini) [45].

Earlier laboratory studies have shown that maize is tolerant to the caraway oil up to the seedling phase [3]. However, the current research results are inconsistent with previously published laboratory results. Even though the initial emergence of maize was similar to the control, up to 0.7 and 0.9 g of MCEO per pot (equal to 69 and 96 g m^−2^), later in the 3-leaf phase of maize significant inhibition was already recorded at 0.4 g of MCEO per pot (equal to 44 g m^−2^). Apart from the effect of the oil dose on the maize’s growth inhibition, the obtained result could also be influenced by the plant’s exposure time to the oil-up to the 3-leaf phase. At this stage, the most sensitive to the effects of MCEO were the roots, which, in contact with the soil-applied MCEO, were of a significantly reduced length and biomass. As a result of impaired roots growth, reduced water uptake by the roots and a reduced elongation of the leaves was noted. The Pearson correlation analysis confirmed this finding, as a high correlation was observed between the biomass and length of the roots and the length of leaves. The hierarchy of traits sensitivity to the MCEO showed that the most affected was the youngest, 3rd leaf length. Analogous changes in maize growth were found in previous pot studies where microencapsulated peppermint oil was applied to three different soil substrates. In the cited studies, the lowest dose of microencapsulated peppermint oil (36 g m^−2^) also inhibited maize cultivars’ elongation and biomass accumulation [37].

Our results also confirmed a correlation between maize growth inhibition and relative chlorophyll content in the leaves (SPAD). However, deviations were noted in two maize cultivars. In the case of cv. Kurant, both SPAD and photosynthetic efficiency, measured by two chlorophyll *a* fluorescence parameters (Fv/Fm and Fv/F0), were maintained at the control level up to the dose of 0.7 g of MCEO per pot. We speculate that the reduced growth parameters in the case of this cultivar did not correlate with the deterioration of the physiological parameters. To the contrary, in the cv. Rywal, despite high SPAD values up to the dose of 1.2 g per pot, the Fv/Fm and Fv/F0 parameters were significantly reduced already at the dose of 0.7 g of MCEO per pot. Many authors observed the correlation of SPAD and Fv/Fm parameter [46,47,48]. In the research of [46], an inverse relationship in tomato leaves was found, namely high photosynthesis efficiency despite the low relative content of chlorophyll in the leaves. According to the authors, this results from acclimation to the low light condition and high light-utilization efficiency for photosynthesis. To the contrary, Ref. [49] showed that the Fv/Fm parameter has a stronger relationship with the Rubisco content than the SPAD readings. Concerning our research, the parameters of photosystem II efficiency (Fv/Fm and Fv/F0) are more sensitive indicators of stress than the SPAD readings, which was confirmed by the PCA analysis.

Moreover, our results also showed that the other tested parameters of the photosystem II state, i.e., the emission spectra F690/F735, F450/F690, F450/735, and PSIIA/C were the least responsive to the changes caused in maize by the MCEO. In the control, maize leaves were characterized by higher values of these ratios than those treated with MCEO. Perhaps it is associated with an increase in carbohydrate content that enables the energy-costly synthesis of secondary metabolites [50], i.e., derivatives of cinnamic, ferulic, sinapic, and caffeic acids correlated with the blue fluorescence [51,52].

## 4. Materials and Methods

### 4.1. Chemical Analysis of the Microencapsulated Caraway Oil

Microencapsulated caraway essential oil (MCEO) used in this experiment was purchased in 2017 from the commercial producer (Hoffmann Aroma, Zamysłowo, Poland). The MCEO was obtained by the method of dry spraying. The carrier for the EO was maltodextrin with a small addition (4.5%) of gum Arabic E414.

The chemical analyses of the MCEO were performed in the laboratory of the Institute of Natural Products and Cosmetics, Lodz University of Technology. The content of caraway EO in the microcapsules was measured three times by the hydrodistillation method (10 g of microcapsules and 100 mL of water) for two hours, using a Clevenger-type apparatus. The volume of the separated EO was multiplied by the specific density of the microcapsules, determined by the pycnometer method. The essential oil was analyzed by gas chromatography coupled with mass spectrometry (GC-FID-MS), using a Trace GC Ultra gas chromatograph coupled with DSQ II mass spectrometer (Thermo Electron Corporation, Waltham, MA, USA). The operating conditions were as follows: non-polar capillary column Rtx-1 ms (60 m × 0.25 mm, 0.25 m film thickness), programmed temperature: 50 (3 min) −300 °C, 4 °C/min. injector (SSL) temperature 280 °C, detector (FID) temperature 300 °C, transfer line temperature 250 °C, carrier gas–helium, flow with constant pressure 200 kPa, split ratio 1:20. The mass spectrometer parameters: ion source temperature 200 °C, ionization energy 70 eV (EI), scan mode: full scan, mass range 33–420. The percentages of constituents were computed from the GC peak area without using a correction factor. Identification of the components was based on a comparison of their mass spectra and linear retention indices (RI, non-polar column), determined regarding a series of n-alkanes C8-C24, compared to those in Adams [53] and computer libraries: NIST 2011, and MassFinder 4.1 (Detlev Hochmuth, Hamburg, Germany).

### 4.2. Description of the Pot Experiments

Two series of dose–response pot experiments were set up between July 2021–February 2021 in a randomized design with four replications in the greenhouse of the Pedagogical University of Krakow. The photoperiod was 14/10, and the temperature was in a range of 5–15 °C at night and 20–30 °C during the day. The bottom of each pot (1 L vol.) was lined with a standard filter paper layer to prevent losses of substrates and the MCEO and next filled up with a certified, pure, yellow sand of grains of 0.2 mm size (BIOVITA, PL). Different doses of MCEO: 0 (control); 0.42; 0.66; 0.91; 1.21 and 1.82 g per pot (equal to 44; 69; 96; 127 and 192 g m^−2^), were mixed with the sand in the pots up to 3-cm-deep. On the same day, two kernels of maize per pot were seeded at a ca. 2.0 cm depth. There were four maize (*Zea mays* L.) cultivars tested in the experiment, i.e., ‘Kurant’ (KU), ‘Pomerania’ (PM), ‘Podole’ (PO), and ‘Rywal’ (RY) (all provided by the breeder HR Smolice Sp. z o. o. Grupa IHAR, PL). Maize was watered with tap water every 2–3 days. No fertilization was applied to prevent a potential interaction between fertilizer and the MCEO. The emergence time of maize was recorded. Both experiments were terminated when each plant reached the three-leaf growth stage (BBCH 13). Based on our previous pot results, that maltodextrin up to a dose of 145 g m^−2^ was neutral to the growth of maize in vermiculite, sandy soil, and clayey soil [37], we did not include the maltodextrin treatment in the present study.

### 4.3. Relative Chlorophyll Content and Chlorophyll Fluorescence Measurements

In the BBCH 13 stage, a relative chlorophyll content analysis in the SPAD values was tested using a Minolta SPAD 502PDL (Konica-Minolta Co., Ltd., Tokyo, Japan) chlorophyll meter on the adaxial side of the 2nd maize leaf. The measurements were made on five plants per treatment.

On the same day, the photosystem II (PSII) performance of maize plants was measured by the selected parameters of chlorophyll *a* fluorescence [33]. The second leaf of each maize plant was acclimated to the dark for 30 min using specialized clips. Then, the leaves were exposed to excitation light of intensity 600 μmol × m^−2^ × s^−1^ by a fluorimeter (Fluorescence Monitoring System–1, Hansatech Instruments Ltd., Norfolk, UK). The measured values were: F0–minimal chlorophyll fluorescence and Fm–maximal chlorophyll fluorescence. Two parameters were calculated: Fv/Fm–the maximum quantum efficiency of PSII and Fv/F0–the maximum primary yield of the photochemistry of PSII. For a healthy sample, the Fv/Fm ratio is around 0.83 and lowers as plant stress increases, reaching 0.3 at the end of senescence [54].

The blue-green and red fluorescence emission spectra on the spectrofluorometer (Perkin-Elmer LS55B, Oswestry, UK) were measured according to [55]. The fluorescence intensity in the range of blue-green light (430–650 nm) was performed at 390 nm excitation and near and far red (650–800 nm), with blue 430 nm excitation. The slot for the excitation radius was 15 nm and for the emitted 20 nm. Four spectra coefficients were calculated: the F690/F735, referring to the fluorescence of chlorophyll in the red and far-red spectrum with a maximum at 690 and 735 nm; the F450/F690 referring to blue/red spectrum with a maximum at 450 and 690 nm; and the F450/F735 referring to blue/far-red spectrum with a maximum at 450 and 735 nm [52,55,56]. The activities of the cortical (C) and antenna (A) parts of the PSII system (the PSIIA/C parameter) were determined according to [57], with peaks at 685 nm and 695 nm indicating A and C of PSII. The results were analyzed using FL WinLab version No. 3.00 (PerkinElmer Life and Analytical Sciences, Waltham, MA, USA).

### 4.4. Morphometric Measurements of Maize

The plants were removed from the pots, cut at the stem’s base. Roots were carefully washed under tap water and gently dried using paper tissues. The length of three fully developed leaf blades and the length of roots were measured with a ruler. Next, shoots and roots of maize were dried in the oven at 105 °C for 24 h. Their dry weight was weighed using laboratory balance (with 0.01 g accuracy).

### 4.5. Statistical Analysis

The normality of the distribution of the nine traits, e.g., length of 1st, 2nd and 3rd leaf, root length, shoot fresh mass, fresh root mass, SPAD, Fv/Fm, Fv/F0, F690/F735, F450/F690, F450/F735, and PSIIA/C was tested with Shapiro–Wilk’s normality test to check whether the analysis of variance (ANOVA) met the assumption that the ANOVA model residuals followed a normal distribution. The homogeneity of variance was tested using Bartlett’s test. Box’s M test tested multivariate normality and homogeneity of variance-covariance matrices. All the traits had a normal distribution. A two-way (cultivar, dose per pot) multivariate analysis of variance (MANOVA) was performed. Following this, two-way analyses of variance (ANOVA) were performed to verify the null hypotheses of a lack of cultivar and dose per pot effects and cultivar × dose per pot interaction effect in terms of the values of the nine observed traits, independently for each trait. The arithmetic means and standard deviations were calculated.

Moreover, Fisher’s least significant differences (LSDs) were estimated at a significance level of α = 0.05. The relationships between the observed traits were estimated based on the cultivar and dose per pot combinations’ means using Pearson’s correlation coefficients. The results were also analyzed using multivariate methods. A principal components analysis (PCA) was applied to present a multi-trait assessment of the similarity of the tested cultivar and dose per pot combinations in a lower number of dimensions with the least possible loss of information. The GenStat v. 18 statistical software package (VSN International) was used for the analyses.

## 5. Conclusions

The pre-emergence, sand-applied caraway essential oil coated in maltodextrin (MCEO) displays a dose-dependent phytotoxic effect on maize. The MCEO effects are visible as both growth reduction of maize, a lower content of chlorophyll in the leaves (measured in SPAD values), and a decrease of chlorophyll *a* fluorescence parameters (Fv/Fm and Fv/F0), which all point to plant stress caused by the MCEO. In conclusion, maize is susceptible to the pre-emergence, sand-applied MCEO at doses 44–192 g m^−2^. Further studies should be undertaken to assess the effects of the different timing of MCEO application, and at lower doses, on the growth of both maize and accompanying weeds.

## Figures and Tables

**Table 1:** Content of volatile constituents in microencapsulated *Carum carvi* L. essential oil.

Compound	RI Lit. 1	RI Exp. 2	Analyzed EO [%]	EO According to [38] [%]	EO According to [39] [%]
α-Thujene	926		t		
*p*-Cymene	1015	1011	t		
Limonene	1025	1023	15.2	30–45	9.8–53.9
Myrcene	1063		-	0.1–1.0	0.1–0.4
*p*-Cymen	1075		t		t
Linalool	1086	1084	t		
*cis-p*-Menth-2,8-dien-1-ol	1104	1104	t		0–0.6
*trans-p*-Menth-2,8-dien-1-ol	1113	1116	0.4		0–0.6
cis-Limonene oxide	1126	1120	0.1		
β-Terpineol	1137	1140	t		
*cis*-Dihydrocarvone	1172	1172	0.8		
*trans*-Dihydrocarvone	1177	1178	0.6	0–2.5	
γ-Terpineol	1177	1182	0.3		
Dihydrocarveol (isomer)	1193	1188	0.4		0–0.1
*trans*-Carveol	1200			0–2.5	
Dihydrocarveol (isomer)	1212	1212	0.2		0–0.1
Carvone	1215	1235	79.9	50–65	43.4–80.2
*trans*-Carvone epoxide	1261	1252	1.0		
Perilla alcohol	1280	1279	0.1		0–0.1

^1^ RI lit—retention index according to literature; ^2^ RI exp—determined retention index.

**Table 2:** Two-way analysis of variance for the analyzed traits of maize.

Trait	Source of Variation	Cultivar	Dose Per Pot	Cultivar × Dose Per Pot	Residual
Length of leaf 1	d.f. ^1^	3	5	15	249
	m.s. ^2^	35.28 ***	406.3 ***	9.37 ***	2.099
Length of leaf 2	d.f.	3	5	15	250
	m.s.	412.15 ***	3032.3 ***	73.10 ***	17.77
Length of leaf 3	d.f.	3	5	15	250
	m.s.	797.19 ***	8204.1 ***	145.08 ***	47.78
Length of roots	d.f.	3	5	15	250
	m.s.	562.1 ***	11964.9 ***	262.9 ***	64.14
Shoot fresh mass	d.f.	3	5	15	235
	m.s.	0.58	30.84 ***	0.419	0.257
Root fresh mass	d.f.	3	5	15	302
	m.s.	0.657	56.80 ***	1.810 ***	0.511
SPAD	d.f.	3	5	15	120
	m.s.	436.6 ***	4778.2 ***	448.5 ***	7.274
Fv/Fm	d.f.	3	5	15	360
	m.s.	0.267 **	5.897 ***	0.381 ***	0.052
Fv/F0	d.f.	3	5	15	360
	m.s.	13.15 ***	156.07 ***	10.69 ***	1.371
F690/F735	d.f.	3	5	15	96
	m.s.	5.753 ***	36.84 ***	6.909 ***	0.0119
F450/F690	d.f.	3	5	15	96
	m.s.	2.423 ***	8.893 ***	1.591 ***	0.058
F450/F735	d.f.	3	5	15	96
	m.s.	11.45 ***	55.92 ***	10.51 ***	0.484
PSIIA/C	d.f.	3	5	15	96
	m.s.	3.701 ***	11.97 ***	2.707 ***	0.005

^1^ d.f.—number of degree of freedom; ^2^ m.s.—mean square. ** *p* < 0.01; *** *p* < 0.001.

**Table 3:** Mean values and standard deviations (s.d.) of biometrical traits of maize for cultivars, dose per pot and cultivar × dose per pot interaction.

Cultivar ^1^	Dose per Pot ^2^	Length Leaf 1		Length Leaf 2		Length Leaf 3		Length Root		Shoot Fresh Mass		Root Fresh Mass	
		Mean	s.d.	Mean	s.d.	Mean	s.d.	Mean	s.d.	Mean	s.d.	Mean	s.d.
KU	0	6.47	1.42	16.5	3.87	26.8	6.62	31.2	11.2	1.66	0.53	2.44	0.94
	0.4	5.59	0.73	14.6	2.1	24.3	2.75	30.0	7.01	1.59	0.58	1.73	0.63
	0.7	3.62	1.43	9.11	3.57	15.0	7.32	14.1	8.04	0.94	0.61	1.29	0.77
	0.9	0.63	1.41	2.10	4.91	2.16	4.94	1.77	4.21	0.26	0.55	0.15	0.25
	1.2	0.71	1.58	2.78	6.58	2.87	6.52	1.91	5.10	0.18	0.40	0.16	0.41
	1.8	0.34	1.12	0.89	2.95	1.19	3.95	1.12	3.71	0.07	0.22	0.07	0.28
PM	0	6.72	0.86	19.5	3.01	26.5	6.00	27.7	8.21	1.34	0.25	2.12	0.69
	0.4	6.30	0.48	18.0	1.55	29.2	5.13	26.8	8.61	1.62	0.37	1.97	0.62
	0.7	3.89	2.73	10.4	7.49	18.5	13.75	14.3	10.1	1.07	0.92	1.61	1.08
	0.9	3.41	2.53	8.25	7.52	10.9	12.2	7.77	7.29	0.63	0.62	1.21	0.89
	1.2	0.43	1.42	1.01	3.35	0.96	3.20	0.84	2.77	0.04	0.12	0.08	0.21
	1.8	0	0	0	0	0	0	0	0	0	0	0	0
PO	0	6.07	0.80	18.1	1.85	28.9	5.68	41.7	13.36	1.72	0.28	1.85	0.37
	0.4	5.58	0.88	16.3	2.09	26.1	2.99	26.8	7.55	1.49	0.53	2.00	0.53
	0.7	4.37	0.78	13.5	3.41	23.5	5.81	22.2	5.76	1.16	0.80	1.86	1.29
	0.9	2.15	2.52	6.07	7.00	10.53	12.15	5.29	6.35	0.75	0.84	0.97	1.20
	1.2	0.34	1.07	1.67	3.83	2.00	5.13	0.76	1.9	0.11	0.26	0.17	0.39
	1.8	0	0	0	0	0	0	0	0	0	0	0	0
RY	0	3.35	2.13	8.98	5.68	16.7	10.1	20.8	15.1	1.34	0.42	1.85	0.64
	0.4	4.62	2.22	11.4	5.53	18.6	9.05	25.6	14.0	1.38	0.23	2.39	0.50
	0.7	3.57	1.86	8.55	5.65	15.2	10.4	16.9	10.8	1.20	1.07	2.15	1.64
	0.9	0.48	1.08	1.53	3.47	2.03	4.53	1.15	2.58	0.09	0.19	0.16	0.35
	1.2	0.22	0.72	0.61	2.02	1.14	3.77	0.91	2.56	0.07	0.20	0.21	0.48
	1.8	0	0	0	0	0	0	0	0	0	0	0	0
LSD_0.05_		0.98		2.838		4.654		5.393		0.341		0.481	
Mean	KU	3.03	2.85	8.01	7.55	12.6	12.2	14.1	15.1	0.85	0.83	1.08	1.11
	PM	3.59	3.07	9.94	8.89	14.9	14.0	13.5	13.4	0.84	0.78	1.24	1.07
	PO	3.28	2.71	9.73	7.96	15.9	13.2	17.2	17.3	0.95	0.83	1.24	1.11
	RY	2.45	2.41	5.33	6.24	9.21	10.8	11.3	14.3	0.68	0.79	1.13	1.27
LSD_0.05_		0.4		1.175		1.927		2.232		0.141		0.199	
Mean	0	5.65	1.93	15.7	5.56	24.7	8.55	30.3	14.1	1.53	0.41	2.12	0.75
	0.4	5.52	1.36	15.1	3.96	24.6	6.62	27.3	9.50	1.53	0.45	1.96	0.62
	0.7	3.88	1.78	10.5	5.43	18.2	10.0	17.0	9.11	1.10	0.83	1.68	1.19
	0.9	1.67	2.27	4.49	6.40	6.42	9.93	3.99	5.90	0.43	0.64	0.64	0.88
	1.2	0.43	1.22	1.51	4.21	1.74	4.72	1.10	3.23	0.10	0.26	0.15	0.37
	1.8	0.08	0.56	0.22	1.48	0.30	1.97	0.28	1.85	0.02	0.11	0.06	0.20
LSD_0.05_		0.5		1.45		2.37		2.75		0.174		0.245	

^1^ Maize cultivar: KU—Kurant; PM—Pomerania; PO—Podole; RY—Rywal. ^2^ Doses (g per pot): 0, 0.4, 0.7, 0.9, 1.2 and 1.8 are equal to (g m^−2^): 44, 69, 96, 127 and 192, respectively.

**Table 4:** Mean values and standard deviations (s.d.) of relative chlorophyll content (SPAD) and fluorescence parameters of maize for cultivars, dose per pot and cultivar × dose per pot interaction.

Cultivar ^1^	Dose per Pot ^2^	SPAD		Fv/Fm		Fv/F0		F690/F735		F450/F690		F450/F735		PSIIA/C	
		Mean	s.d.	Mean	s.d.	Mean	s.d.	Mean	s.d.	Mean	s.d.	Mean	s.d.	Mean	s.d.
KU	0	21.90	2.34	0.80	0.02	4.01	0.60	2.28	0.30	1.35	0.33	3.08	0.98	1.50	0.07
	0.4	21.10	3.69	0.81	0.03	4.37	0.70	2.10	0.09	0.98	0.28	2.06	0.64	1.53	0.09
	0.7	21.20	3.98	0.80	0.025	4.05	0.54	2.64	0.57	1.19	0.44	2.95	0.37	1.54	0.06
	0.9	6.22	9.67	0.11	0.28	0.45	1.17	2.35	0.32	1.14	0.21	2.6	0.35	1.50	0.04
	1.2	0	0	0.11	0.28	0.48	1.23	2.06	0.24	1.08	0.17	2.25	0.56	1.46	0.04
	1.8	0	0	0.06	0.21	0.25	0.95	2.24	0.11	0.90	0.09	2.03	0.18	1.54	0.05
PM	0	26.60	1.77	0.80	0.01	4.15	0.27	1.95	0.07	1.42	0.12	2.78	0.21	1.53	0.04
	0.4	23.30	3.16	0.66	0.31	3.29	1.54	2.32	0.28	0.92	0.23	2.09	0.41	1.53	0.09
	0.7	18.60	1.63	0.61	0.33	2.83	1.63	2.73	0.38	0.91	0.30	2.45	0.75	1.49	0.09
	0.9	15.80	1.40	0.73	0.21	3.49	1.22	2.91	0.58	0.98	0.15	2.90	1.05	1.45	0.03
	1.2	0	0	0.23	0.38	1.18	1.94	0	0	0	0	0	0	0	0
	1.8	0	0	0	0	0	0	0	0	0	0	0	0	0	0
PO	0	23.40	2.27	0.82	0.01	4.57	0.18	2.13	0.25	0.87	0.16	1.85	0.38	1.56	0.06
	0.4	17.80	1.31	0.81	0.01	4.40	0.47	2.44	0.17	1.35	0.41	3.24	0.82	1.45	0.06
	0.7	16.00	2.15	0.79	0.03	3.94	0.58	2.77	0.65	1.25	0.28	3.44	1.09	1.50	0.08
	0.9	15.50	1.23	0.46	0.41	2.32	2.10	2.89	0.37	1.27	0.10	3.66	0.46	1.49	0.06
	1.2	13.30	1.24	0.64	0.35	3.57	1.95	2.27	0.32	0.98	0.34	2.15	0.51	1.50	0.10
	1.8	0	0	0	0	0	0	0	0	0	0	0	0	0	0
RY	0	20.8	1.94	0.79	0.02	3.83	0.51	2.45	0.35	1.28	0.16	3.12	0.38	1.43	0.11
	0.4	21.00	2.60	0.79	0.01	3.78	0.29	2.51	0.72	1.40	0.42	3.64	2.02	1.50	0.11
	0.7	19.80	1.76	0.56	0.37	2.69	1.84	2.37	0.46	1.30	0.12	3.12	0.76	1.46	0.07
	0.9	22.20	0.14	0.43	0.39	1.82	1.70	2.19	0.26	0.77	0.16	1.67	0.32	1.53	0.09
	1.2	22.60	0.30	0.46	0.41	2.28	2.05	2.14	0.07	0.94	0.31	1.99	0.60	1.49	0.06
	1.8	0	0	0	0	0	0	0	0	0	0	0	0	0	0
LSD_0.05_		1.826		0.154		0.787		0.234		0.163		0.472		0.046	
Mean	KU	11.70	10.90	0.49	0.39	2.50	2.06	2.28	0.35	1.11	0.29	2.49	0.67	1.51	0.06
	PM	14.00	10.80	0.53	0.38	2.61	1.90	1.65	1.26	0.70	0.56	1.70	1.35	1.00	0.72
	PO	14.30	7.37	0.62	0.35	3.28	1.90	2.08	1.04	0.95	0.52	2.39	1.41	1.25	0.57
	RY	17.70	8.22	0.53	0.37	2.53	1.81	1.94	0.96	0.95	0.53	2.25	1.50	1.23	0.57
LSD_0.05_		0.756		0.064		0.326		0.097		0.068		0.196		0.019	
Mean	0	23.20	2.96	0.80	0.02	4.14	0.50	2.20	0.31	1.23	0.29	2.70	0.74	1.50	0.08
	0.4	20.80	3.31	0.77	0.16	3.96	0.98	2.34	0.40	1.16	0.39	2.76	1.28	1.50	0.09
	0.7	18.90	3.08	0.69	0.27	3.39	1.40	2.63	0.51	1.16	0.32	2.99	0.81	1.50	0.08
	0.9	14.90	7.41	0.43	0.39	2.02	1.90	2.58	0.49	1.04	0.24	2.71	0.93	1.49	0.02
	1.2	8.98	9.79	0.36	0.40	1.88	2.13	1.62	0.98	0.75	0.50	1.60	1.05	1.11	0.66
	1.8	0	0	0.01	0.10	0.06	0.48	0.56	1.00	0.226	0.40	0.51	0.90	0.38	0.68
LSD_0.05_		0.931		0.078		0.402		0.119		0.083		0.241		0.023	

^1^ Maize cultivar: KU—Kurant; PM—Pomerania; PO—Podole; RY—Rywal. ^2^ Doses (g per pot): 0, 0.4, 0.7, 0.9, 1.2 and 1.8 are equal to (g m^−2^): 44, 69, 96, 127 and 192, respectively.

**Table 5:** Pearson’s correlation coefficients between all pairs of analyzed traits of maize (r_0.001_ = 0.6304).

Trait	Fv/Fm	Fv/F0	Leaf 1	Leaf 2	Leaf 3	Root Length	Shoot Fresh Mass	SPAD	Root Fresh Mass	F690/F735	F450/F690	F450/F735
Fv/F0	0.99 ***											
Leaf/1	0.84 ***	0.84 ***										
Leaf/2	0.82 ***	0.83 ***	0.99 ***									
Leaf/3	0.83 ***	0.84 ***	0.99 ***	0.99 ***								
Root length	0.8 ***	0.82 ***	0.95 ***	0.95 ***	0.96 ***							
Shoot fresh mass	0.84 ***	0.84 ***	0.97 ***	0.95 ***	0.98 ***	0.97 ***						
SPAD	0.89 ***	0.86 ***	0.75 ***	0.73 ***	0.74 ***	0.71 ***	0.75 ***					
Root fresh mass	0.85 ***	0.83 ***	0.94 ***	0.92 ***	0.94 ***	0.91 ***	0.96 ***	0.76 ***				
F690/F735	0.67 ***	0.64 ***	0.5 *	0.49 *	0.5 *	0.41 *	0.53 **	0.67 ***	0.54 **			
F450/F690	0.71 ***	0.68 ***	0.61 **	0.6 **	0.6 **	0.54 **	0.64 ***	0.69 ***	0.68 ***	0.9 ***		
F450/735	0.7 ***	0.67 ***	0.58 **	0.56 **	0.57 **	0.49 *	0.62 **	0.65 ***	0.68 ***	0.92 ***	0.97 ***	
PSIIA/C	0.66 ***	0.64 ***	0.52 **	0.52 ***	0.52 **	0.47 *	0.54 **	0.71 ***	0.52 **	0.96 ***	0.91 ***	0.86 ***

Maize cultivar: KU–Kurant; PM–Pomerania; PO–Podole; RY–Rywal. Doses of MCEO (g per pot): 0, 0.4, 0.7, 0.9, 1.2 and 1.8, are equal to (g m^−2^): 44; 69; 96; 127 and 192, respectively. * *p* < 0.05; ** *p* < 0.01; *** *p* < 0.001.

## Data Availability

The data presented in this study are available on request from the corresponding author.

## Sample Availability

Samples of the MCEO are available on request from the corresponding author.
